# Supplementary material for: Calciprotein Particles Cause Physiologically Significant Pro-Inflammatory Response in Endothelial Cells and Systemic Circulation
Source: Int J Mol Sci. 2022 Nov 29;23(23):14941. doi: 10.3390/ijms232314941 (PMC9738209; doi:10.3390/ijms232314941)
Supplement: Supplementary file 1 [file ijms-23-14941-s001.zip › ijms-2006269-supplementary.pdf]

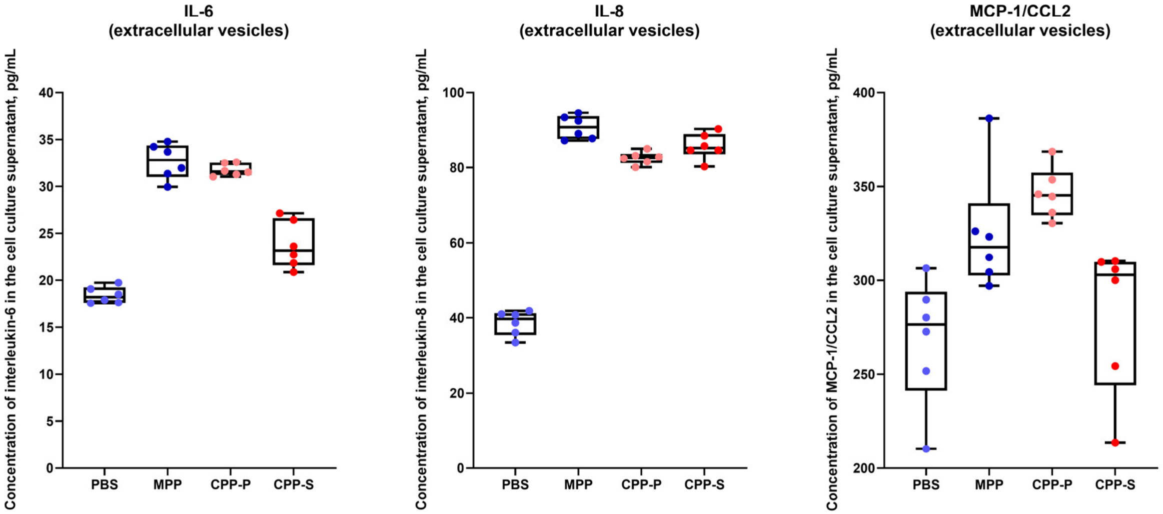

Figure S1. Cytokine profiling of the conditioned medium from intact HCAEC treated with extracellular vesicles from HCAEC incubated with MPPs, CPP-P, CPP-S, or control PBS solution for 24 hours. Specific enzyme-linked immunosorbent assay kits for the measurement of IL-6 (left), IL-8 (center), and MCP-1/CCL2 (right). Each dot on the plots represents one measurement (n = 6 measurements per group). Whiskers indicate the range, box bounds indicate the 25th–75th percentiles, and centre lines indicate the median.

Table S1. Complete blood count measurements of the human blood co-incubated with MPPs, CPP-P, CPP-S, or control physiological saline solution (PS, 0.9% NaCl) for 0.5, 2, 4, or 24 hours.

**Complete blood count parameter**	**0 min**	**30 minutes**					**2 hours**					**4 hours**					**24 hours**				
		PS	MPP	CPP-P	CPP-S	*p*	PS	MPP	CPP-P	CPP-S	*p*	PS	MPP	CPP-P	CPP-S	*p*	PS	MPP	CPP-P	CPP-S	*p*
Human blood																					
White blood cell count (WBC)	9.3	8.9	9.0	9.0	9.0	0.9	9.0	9.2	8.8	9.0	0.8	9.0	9.0	8.8	9.0	0.9	8.2	9.0	9.0	8.8	0.1
Neutrophil count (NE#)	4.8	4.7	4.5	4.7	4.5	0.7	4.5	4.5	4.5	4.5	0.5	4.7	4.7	4.7	4.7	0.9	4.5	5.2	4.7	4.5	0.2
Neutrophil percentage (NE%)	51	52	50	52	51	0.8	51	50	51	51	0.9	51	52	50	52	0.9	53	57	54	52	0.3
Lymphocyte count (LY#)	3.5	3.4	3.6	3.2	3.0	0.4	3.4	3.5	3.2	3.2	0.6	3.2	3.5	3.5	3.2	0.5	3.0	2.8	3.0	3.0	0.7
Lymphocyte percentage (LY%)	36	36	37	35	36	0.8	36	36	36	35	0.9	35	35	37	35	0.8	34	32	34	36	0.8
Monocyte count (MO#)	1.0	1.0	1.0	1.0	1.1	0.7	1.0	1.0	1.1	1.0	0.8	1.1	1.1	1.1	1.1	0.9	1.0	0.9	1.0	0.9	0.9
Monocyte percentage (MO%)	10	10	10	10	11	0.9	10	10	11	10	0.9	11	11	11	11	0.8	10	9	10	9	0.2
Red blood cell count (RBC)	4.7	4.6	4.5	4.6	4.5	0.6	4.5	4.5	4.5	4.4	0.7	4.6	4.6	4.5	4.5	0.2	4.6	4.7	4.7	4.6	0.1
Haemoglobin (HGB)	145	137	138	139	135	0.8	135	135	135	135	0.7	138	139	137	135	0.6	139	140	139	138	0.7
Haematocrit (HCT)	44	43	43	43	42	0.8	43	44	43	43	0.9	44	44	44	44	0.7	47	49	48	48	0.4

Mean corpuscular volume (MCV)	92	94	94	95	95	0.8	95	96	96	96	0.9	96	97	97	97	0.7	102	105	102	103	0.1
Mean corpuscular hemoglobin (MCH)	30.2	30.1	30.1	30.1	30.3	0.5	30.0	29.9	30.0	30.4	0.3	30.0	30.3	29.9	30.2	0.3	30.4	30.0	29.8	30.1	0.3
Mean corpuscular hemoglobin concentration (MCHC)	330	319	322	318	320	0.9	316	311	311	317	0.7	309	311	307	312	0.5	293	285	290	293	0.1
Red cell distribution width (RDW-CV)	12.2	12.4	12.2	12.3	12.3	0.9	12.4	12.3	12.2	12.4	0.6	12.5	12.5	12.5	12.5	0.7	14.0	14.5	13.8	14.0	0.3
Platelet count (PLT)	246	242	243	240	248	0.4	236	235	240	235	0.8	242	228	228	235	0.5	233	230	228	228	0.4
Plateletcrit (PCT)	0.26	0.26	0.25	0.25	0.29	0.2	0.25	0.25	0.25	0.25	0.8	0.25	0.25	0.25	0.25	0.3	0.25	0.25	0.25	0.25	0.9
Mean platelet volume (MPV)	11.1	11.2	11.1	11.2	11.1	0.9	10.9	11.3	11.0	11.0	0.5	10.6	10.5	10.5	10.7	0.3	10.7	11.0	10.5	10.7	0.3
Platelet distribution width (PDW)	13.6	12.8	12.6	12.7	12.7	0.9	12.5	12.6	12.6	12.5	0.9	11.9	11.4	11.7	11.5	0.6	12.3	12.0	11.4	10.9	0.1

Table S2. Primers for the quantitative reverse transcription polymerase chain reaction and its parameters.

Gene	Forward primer	Reverse primer
	Cell adhesion molecules	
VCAM1	5′-CGTCTTGGTCAGCCCTTCCT-3′	5′-ACATTCATATACTCCCGCATCCTTC-3′
ICAM1	5′-TTGGGCATAGAGACCCCGTT-3′	5′-GCACATTGCTCAGTTCATACACC-3′
SELE	5′-GCACAGCCTTGTCCAACC-3′	5′-ACCTCACCAAACCCTTCG-3′
SELP	5′-ATGGGTGGGAACCAAAAAGG-3′	5′-GGCTGACGGACTCTTGATGTAT-3′
	Pro-inflammatory cytokines	
IL6	5′-GGCACTGGCAGAAAACAACC-3′	5′-GCAAGTCTCCTCATTGAATCC-3′
CXCL8	5′-CAGAGACAGCAGAGCACAC-3′	5′-AGTTCTTTAGCACTCCTTGGC-3′
CCL2	5′-TTCTGTGCCTGCTGCTCATAG-3′	5′-AGGTGACTGGGGCATTGATTG-3′
CXCL1	5′-GCTTGCCTCAATCCTGCATCC-3′	5′-ACAATCCAGGTGGCCTCTGC-3′
MIF	5′-GGTGTCCGAGAAGTCAGGCA-3′	5′-GGGGCACGTTGGTGTTTACG-3′
	NO synthase	
NOS3	5′-GTGATGGCGAAGCGAGTGAAG-3′	5′-CCGAGCCCGAACACACAGAAC-3′
	Endothelial-to-mesenchymal transition	
SNAI1	5′-CAGACCCACTCAGATGTCAAGAA-3′	5′-GGGCAGGTATGGAGAGGAAGA-3′
SNAI2	5′-ACTCCGAAGCCAAATGACAA-3′	5′-CTCTCTCTGTGGGTGTGTGT-3′
TWIST1	5′-GTCCGCAGTCTTACGAGGAG-3′	5′-GCTTGAGGGTCTGAATCTTGCT-3′
ZEB1	5′-GATGATGAATGCGAGTCAGATGC-3′	5′-ACAGCAGTGTCTTGTTGTTGT-3′
CDH5	5′-AAGCGTGAGTCGCAAGAATG-3′	5′-TCTCCAGGTTTTCGCCAGTG-3′
CDH2	5′-GCTTCTGGTGAAATCGCATTA-3′	5′-AGTCTCTCTTCTGCCTTTGTAG-3′
	Arterial to venous transition	
HES1	5′-AGATAGCTCGCGGCATTCCAAG-3′	5′-ACTTCCCCAGCACACTTGGGT-3′
HEY1	5′-GCCGACGAGACCGGATCAAT-3′	5′-GGCGTGCGCGTCAAAGTAA-3′

HEY2	5′-ACCTCCGAGAGCGACATGGA-3′	5′-CGATCCCGACGCCTTTTCTC-3′
NOTCH1	5′-GCTCACGCTGACGGAGTACA-3′	5′-ATGGAAGCTGGGTGGGCAGT-3′
NR2F2	5′-AGAGCAAGTGGAGAAGCTCAAG-3′	5′-TACATCAGAGAGACCACAGGCA-3′
	Housekeeping genes	
PECAM1	5′-TGGCGCATGCCTGTAGTA-3′	5′-TCCGTTTCCTGGGTTCAA-3′

Table S3. Average ΔCt values and their standard deviations (HCAEC).

	HCAEC											
	**Complete conditioned medium**				**Extracellular vesicle-depleted conditioned medium**				**Extracellular vesicles**			
	**PBS**	**MPP**	**CPP-P**	**CPP-S**	**PBS**	**MPP**	**CPP-P**	**CPP-S**	**PBS**	**MPP**	**CPP-P**	**CPP-S**
VCAM1	0.0021 ± 0.0008	0.0012 ± 0.0002	0.0124 ± 0.0003	0.0054 ± 0.0015	0.0024 ± 0.0005	0.0016 ± 0.0003	0.0009 ± 0.0001	0.0013 ± 0.0011	-	-	-	-
	1	0.57	5.90	2.57	1	0.67	0.38	0.54	-	-	-	-
ICAM1	0.0232 ± 0.0049	0.0196 ± 0.0045	0.0221 ± 0.0038	0.0968 ± 0.0136	0.0164 ± 0.0043	0.0127 ± 0.0031	0.0214 ± 0.0009	0.0210 ± 0.0064	0.0096 ± 0.0042	0.0090 ± 0.0020	0.0170 ± 0.0059	0.0599 ± 0.0221
	1	0.84	0.95	4.17	1	0.77	1.30	1.28	1	0.94	1.77	6.23
SELE	0.0007 ± 0.0003	0.0002 ± 0.0001	0.0025 ± 0.0006	0.0035 ± 0.0012	0.0014 ± 0.0003	0.0005 ± 0.00005	0.0002 ± 0.00001	0.0015 ± 0.0001	0.00006 ± 0.00002	0.00013 ± 0.0000003	0.00005 ± 0.00006	0.000014 ± 0.00004
	1	0.28	3.57	5	1	0.36	0.14	1.07	1	2.16	0.83	0.23
SELP	0.0197 ± 0.0060	0.0183 ± 0.0058	0.0319 ± 0.0031	0.0780 ± 0.0177	0.0151 ± 0.0021	0.0081 ± 0.0014	0.0101 ± 0.0007	0.0092 ± 0.0017	0.0328 ± 0.0082	0.0192 ± 0.0077	0.0250 ± 0.0137	0.0584 ± 0.0066
	1	0.92	1.61	3.95	1	0.54	0.67	0.61	1	0.58	0.76	1.78
IL6	0.0048 ± 0.0224	0.0033 ± 0.0011	0.0087 ± 0.0079	0.0260 ± 0.0079	0.0040 ± 0.0009	0.0037 ± 0.0005	0.0034 ± 0.0001	0.0023 ± 0.0020	0.0015 ± 0.0004	0.0048 ± 0.0016	0.0037 ± 0.0034	0.0027 ± 0.0009
	1	0.68	1.81	5.41	1	0.93	0.85	0.58	1	3.2	2.46	1.8
CXCL8	0.0487 ± 0.0224	0.0485 ± 0.0137	0.4233 ± 0.3904	0.2463 ± 0.0544	0.0524 ± 0.0095	0.0521 ± 0.0066	0.1523 ± 0.0014	0.0562 ± 0.0491	0.0092 ± 0.0014	0.0244 ± 0.0061	0.0199 ± 0.0073	0.0116 ± 0.0026
	1	0.99	8.69	5.05	1	0.99	2.91	1.07	1	2.65	2.16	1.26
CCL2	0.0855 ± 0.0310	0.0529 ± 0.0156	0.2691 ± 0.0364	0.2914 ± 0.0749	0.0435 ± 0.0073	0.0396 ± 0.0073	0.0748 ± 0.0055	0.0391 ± 0.0344	0.0222 ± 0.0046	0.0244 ± 0.0077	0.0218 ± 0.0046	0.0117 ± 0.0031
	1	0.61	3.14	3.40	1	0.91	1.72	0.90	1	1.09	0.98	0.52

CXCL1	0.1643 ± 0.0394	0.1051 ± 0.0026	0.4207 ± 0.2015	0.7013 ± 0.2099	0.1247 ± 0.0283	0.1145 ± 0.0093	0.1411 ± 0.0031	0.0718 ± 0.0664	0.0364 ± 0.0140	0.0531 ± 0.0100	0.0856 ± 0.0466	0.0355 ± 0.0049
	1	0.63	2.56	4.26	1	0.92	1.13	0.58	1	1.45	2.35	0.97
MIF	0.3026 ± 0.1241	0.1405 ± 0.0475	0.1518 ± 0.1382	0.5929 ± 0.2086	0.2004 ± 0.0345	0.1166 ± 0.0150	0.0750 ± 0.0098	0.0662 ± 0.0574	0.1529 ± 0.0081	0.2342 ± 0.0975	0.1196 ± 0.0970	0.1598 ± 0.0607
	1	0.46	0.50	1.95	1	0.58	0.37	0.33	1	1.53	0.78	1.04
NOS3	0.0088 ± 0.0029	0.0092 ± 0.0017	0.0024 ± 0.0022	0.0258 ± 0.0056	0.0124 ± 0.0026	0.0050 ± 0.0009	0.0144 ± 0.0005	0.0031 ± 0.0027	0.0005 ± 0.00002	0.00025 ± 0.0001	0.00013 ± 0.0001	0.0001 ± 0.00003
	1	1.04	0.27	2.93	1	0.40	1.16	0.25	1	0.5	0.26	0.2
SNAI1	0.0017 ± 0.0008	0.0104 ± 0.0023	0.0124 ± 0.0024	0.0488 ± 0.0125	0.0113 ± 0.0025	0.0078 ± 0.0007	0.0118 ± 0.0004	0.0032 ± 0.0028	0.0106 ± 0.0037	0.0139 ± 0.0029	0.0141 ± 0.0058	0.0196 ± 0.0023
	1	6.11	7.29	28.70	1	0.69	1.04	0.28	1	1.31	1.33	1.84
SNAI2	0.0001 ± 0.0001	0.0009 ± 0.0002	0.0008 ± 0.0007	0.0011 ± 0.0005	0.0016 ± 0.0003	0.0011 ± 0.0002	0.0006 ± 0.0001	0.0007 ± 0.0006	0.0134 ± 0.0032	0.0172 ± 0.0034	0.0210 ± 0.0086	0.0434 ± 0.0084
	1	9	8	11	1	0.69	0.38	0.44	1	1.28	1.56	3.23
TWIST1	0.00014 ± 0.0001	0.0001 ± 0.00001	0.00014 ± 0.0001	0.0002 ± 0.0001	0.00008 ± 0.00003	0.0001 ± 0.00002	0.00009 ± 0.00001	0.00004 ± 0.00003	0.00035 ± 0.0001	0.00032 ± 0.00004	0.00040 ± 0.0003	0.0014 ± 0.0002
	1	0.71	1	1.42	1	1.25	1.13	0.50	1	0.91	1.14	4
ZEB1	0.0110 ± 0.0049	0.0062 ± 0.0018	0.0098 ± 0.0088	0.0101 ± 0.0029	0.0230 ± 0.0046	0.0101 ± 0.0029	0.0125 ± 0.0016	0.0090 ± 0.0078	0.0298 ± 0.0078	0.0485 ± 0.0197	0.0326 ± 0.0273	0.0464 ± 0.0081
	1	0.56	0.89	0.91	1	0.44	0.54	0.39	1	1.62	1.09	1.55
CDH5	1.5391 ± 0.6716	0.6480 ± 0.1845	0.8264 ± 0.2449	1.4559 ± 0.2875	0.5347 ± 0.0538	0.4014 ± 0.0593	0.2901 ± 0.0321	0.1332 ± 0.1179	0.1422 ± 0.0384	0.1705 ± 0.0395	0.1381 ± 0.0517	0.0429 ± 0.0100
	1	0.42	0.53	0.94	1	0.75	0.54	0.25	1	1.19	0.97	0.30
CDH2	0.0754 ± 0.0276	0.0573 ± 0.0168	0.1463 ± 0.0555	0.0645 ± 0.0131	0.0655 ± 0.0088	0.0568 ± 0.0121	0.0480 ± 0.0008	0.0189 ± 0.0166	0.0245 ± 0.0072	0.0088 ± 0.0040	0.0035 ± 0.0038	0.0066 ± 0.0013

	1	0.75	1.94	0.85	1	0.87	0.73	0.29	1	0.35	0.14	0.26
HES1	0.0161 ± 0.0073	0.0073 ± 0.0026	0.0091 ± 0.0086	0.0432 ± 0.0070	0.0141 ± 0.0024	0.0105 ± 0.0018	0.0118 ± 0.0029	0.0071 ± 0.0062	0.0034 ± 0.0007	0.0038 ± 0.0013	0.0028 ± 0.0022	0.0027 ± 0.0008
	1	0.45	0.56	2.68	1	0.74	0.84	0.50	1	1.11	0.82	0.79
HEY1	0.0006 ± 0.0021	0.0004 ± 0.0001	0.0005 ± 0.0005	0.0011 ± 0.0003	0.0007 ± 0.0001	0.0005 ± 0.0001	0.0004 ± 0.0002	0.0003 ± 0.0003	0.0001 ± 0.00002	0.000027 ± 0.000003	0.0001 ± 0.00002	0.00013 ± 0.00002
	1	0.66	0.83	1.83	1	0.71	0.57	0.43	1	0.27	1	1.3
HEY2	0.0014 ± 0.0004	0.0010 ± 0.0004	0.0005 ± 0.0005	0.0015 ± 0.0005	0.0006 ± 0.0002	0.0007 ± 0.0001	0.00068 ± 0.0001	0.0004 ± 0.0003	0.00037 ± 0.0002	0.00042 ± 0.0001	0.00034 ± 0.0002	0.00029 ± 0.0001
	1	0.71	0.35	1.07	1	1.17	1.13	0.67	1	1.13	0.91	0.78
NOTCH1	0.00032 ± 0.0002	0.00035 ± 0.0001	0.0002 ± 0.0002	0.0008 ± 0.0003	0.0002 ± 0.00001	0.0003 ± 0.00004	0.00021 ± 0.00002	0.0001 ± 0.0001	0.00006 ± 0.00002	0.000034 ± 0.00001	0.000021 ± 0.00002	0.00003 ± 0.00001
	1	1.09	0.62	2.5	1	1.50	1.05	0.50	1	0.56	0.35	0.5
NR2F2	0.1904 ± 0.0556	0.1894 ± 0.0687	0.1762 ± 0.1606	0.2524 ± 0.0751	0.1639 ± 0.0405	0.1606 ± 0.0162	0.0744 ± 0.0203	0.0775 ± 0.0671	0.2163 ± 0.0493	0.4126 ± 0.1523	0.3195 ± 0.2714	0.3240 ± 0.0816
	1	0.99	0.92	1.32	1	0.98	0.45	0.47	1	1.90	1.47	1.49

Table S4. Average ΔCt values and their standard deviations (HITAEC).

	HITAEC											
	**Complete conditioned medium**				**Extracellular vesicle-depleted conditioned medium**				**Extracellular vesicles**			
	**PBS**	**MPP**	**CPP-P**	**CPP-S**	**PBS**	**MPP**	**CPP-P**	**CPP-S**	**PBS**	**MPP**	**CPP-P**	**CPP-S**
VCAM1	0.0065 ± 0.0020	0.0032 ± 0.0008	0.0050 ± 0.0005	0.0045 ± 0.0002	0.0077 ± 0.0015	0.0191 ± 0.0035	0.0161 ± 0.0228	0.0467 ± 0.0099	-	-	-	-
	1	0.49	0.76	0.69	1	2.48	2.09	6.06	-	-	-	-
ICAM1	0.1317 ± 0.0206	0.0685 ± 0.0218	0.0501 ± 0.0434	0.0889 ± 0.0434	0.0488 ± 0.0151	0.0411 ± 0.0151	0.0467 ± 0.0042	0.0424 ± 0.0068	0.0126 ± 0.0070	0.0047 ± 0.0009	0.0099 ± 0.0034	0.0042 ± 0.0011
	1	0.52	0.38	0.67	1	0.84	0.96	0.87	1	0.37	0.78	0.33
SELE	0.0065 ± 0.0025	0.0107 ± 0.0026	0.0088 ± 0.0077	0.0108 ± 0.0015	0.0159 ± 0.0035	0.0145 ± 0.0028	0.0279 ± 0.0284	0.0406 ± 0.0124	0.0050 ± 0.0015	0.0024 ± 0.0004	0.0005 ± 0.0005	0.0043 ± 0.0004
	1	1.64	1.35	1.66	1	0.92	1.75	2.55	1	0.48	0.1	0.86
SELP	0.0039 ± 0.0017	0.0019 ± 0.0004	0.0040 ± 0.0001	0.0043 ± 0.0008	0.0035 ± 0.0008	0.0167 ± 0.0031	0.0134 ± 0.0049	0.0214 ± 0.0069	0.0019 ± 0.0013	0.0025 ± 0.0011	0.0008 ± 0.0003	0.0016 ± 0.0005
	1	0.48	1.02	1.10	1	4.77	3.83	6.11	1	1.31	0.42	0.84
IL6	0.00061 ± 0.0002	0.00057 ± 0.0001	0.0005 ± 0.0005	0.0029 ± 0.0003	0.0009 ± 0.0002	0.00083 ± 0.0001	0.00082 ± 0.0008	0.0027 ± 0.0009	0.0050 ± 0.0020	0.0032 ± 0.0012	0.0032 ± 0.0028	0.0036 ± 0.0003
	1	0.93	0.81	4.75	1	0.92	0.91	3.00	1	0.64	0.64	0.72
CXCL8	0.0405 ± 0.0162	0.0877 ± 0.0240	0.0921 ± 0.0043	0.1023 ± 0.0195	0.0758 ± 0.0168	0.0670 ± 0.0129	0.1903 ± 0.0701	0.1787 ± 0.0575	0.0750 ± 0.0231	0.0519 ± 0.0161	0.0269 ± 0.0239	0.0676 ± 0.0023
	1	2.16	2.27	2.52	1	0.88	2.51	2.36	1	0.69	0.35	0.90
CCL2	0.3029 ± 0.1448	0.1573 ± 0.0483	0.2993 ± 0.0181	0.3437 ± 0.0644	0.1682 ± 0.0371	0.1510 ± 0.0303	0.2311 ± 0.1006	0.2640 ± 0.0915	0.2483 ± 0.0798	0.2611 ± 0.0749	0.1368 ± 0.0021	0.2736 ± 0.0082
	1	0.51	0.98	1.13	1	0.90	1.37	1.57	1	1.05	0.55	1.10

CXCL1	0.1143 ± 0.0538	0.1280 ± 0.0348	0.1108 ± 0.0048	0.1670 ± 0.0292	0.0473 ± 0.0171	0.0592 ± 0.0107	0.1033 ± 0.0456	0.1143 ± 0.0632	0.0577 ± 0.0181	0.0914 ± 0.0550	0.0578 ± 0.0117	0.0620 ± 0.0111
	1	1.11	0.96	1.46	1	1.25	2.18	2.42	1	1.58	1	1.07
MIF	0.2785 ± 0.1114	0.2478 ± 0.0668	0.2209 ± 0.1915	0.4082 ± 0.0789	0.1057 ± 0.0277	0.0791 ± 0.0160	0.1260 ± 0.1221	0.1719 ± 0.0623	0.6260 ± 0.2575	0.4185 ± 0.1443	0.4373 ± 0.3912	0.7639 ± 0.0288
	1	0.88	0.79	1.46	1	0.75	1.19	1.63	1	0.66	0.69	1.22
NOS3	0.0164 ± 0.0071	0.0026 ± 0.0005	0.0042 ± 0.0036	0.0046 ± 0.0014	0.0021 ± 0.0005	0.0037 ± 0.0010	0.0024 ± 0.0023	0.0070 ± 0.0027	0.00026 ± 0.0002	0.00004 ± 0.00003	-	0.00005 ± 0.00003
	1	0.15	0.25	0.28	1	1.76	1.14	3.33	1	0.15	-	0.19
SNAI1	0.0082 ± 0.0026	0.0092 ± 0.0025	0.0148 ± 0.0017	0.0079 ± 0.0012	0.0040 ± 0.0007	0.0026 ± 0.0006	0.0023 ± 0.0022	0.0038 ± 0.0003	0.0092 ± 0.0043	0.0054 ± 0.0018	0.0025 ± 0.0022	0.0057 ± 0.0011
	1	1.12	1.80	0.96	1	0.65	0.58	0.95	1	0.58	0.27	0.61
SNAI2	0.0003 ± 0.0001	0.00022 ± 0.00004	0.0006 ± 0.0001	0.00024 ± 0.0002	0.000018 ± 0.00001	0.00001 ± 0.00001	0.000022 ± 0.00001	0.00004 ± 0.00003	0.0006 ± 0.0003	0.0007 ± 0.0002	0.0010 ± 0.0001	0.0002 ± 0.0001
	1	0.73	2	0.8	1	0.56	1.22	2.22	1	1.16	1.66	0.33
TWIST1	0.00001 ± 0.000001	0.00001 ± 0.00001	0.000003 ± 0.000004	-	0.00001 ± 0.000002	0.000002 ± 0.000001	0.000003 ± 0.000001	0.000004 ± 0.000003	0.00016 ± 0.0001	0.00020 ± 0.00003	0.00011 ± 0.0001	0.0001 ± 0.0001
	1	1	0.3	-	1	0.20	0.30	0.40	1	1.25	0.68	0.62
ZEB1	0.0045 ± 0.0019	0.0070 ± 0.0018	0.0039 ± 0.0034	0.0040 ± 0.0011	0.0097 ± 0.0039	0.0121 ± 0.0034	0.0077 ± 0.0015	0.0193 ± 0.0055	0.0371 ± 0.0118	0.0495 ± 0.0140	0.0196 ± 0.0170	0.0308 ± 0.0017
	1	1.55	0.86	0.88	1	1.25	0.79	1.99	1	1.33	0.52	0.83
CDH5	0.6622 ± 0.3683	1.0498 ± 0.2574	0.6092 ± 0.0339	1.5231 ± 0.2239	0.3708 ± 0.0998	0.5407 ± 0.0932	0.4729 ± 0.1358	0.4295 ± 0.1596	0.0905 ± 0.0353	0.0591 ± 0.0281	0.0289 ± 0.0050	0.0001 ± 0.0001
	1	1.58	0.91	2.30	1	1.46	1.28	1.16	1	0.65	0.31	0.001

CDH2	0.0010 ± 0.0005	0.0013 ± 0.0003	0.0012 ± 0.0001	0.0008 ± 0.0001	0.0014 ± 0.0004	0.0014 ± 0.0001	0.0257 ± 0.0415	0.0015 ± 0.0004	0.00002 ± 0.00002	-	-	0.000024 ± 0.00001
	1	1.3	1.2	0.8	1	1	18.36	1.07	1	-	-	1.2
HES1	0.0322 ± 0.0138	0.0195 ± 0.0040	0.0164 ± 0.0142	0.0266 ± 0.0044	0.0135 ± 0.0034	0.0147 ± 0.0036	0.0084 ± 0.0081	0.0228 ± 0.0071	0.0079 ± 0.0036	0.0087 ± 0.0029	0.0048 ± 0.0042	0.0045 ± 0.0006
	1	0.60	0.50	0.82	1	1.09	0.62	1.69	1	1.10	0.60	0.56
HEY1	0.0035 ± 0.0004	0.0031 ± 0.0006	0.0055 ± 0.0013	0.0038 ± 0.0005	0.0026 ± 0.0005	0.0024 ± 0.0006	0.0018 ± 0.0008	0.0028 ± 0.0007	0.00049 ± 0.0001	0.0007 ± 0.0001	0.0002 ± 0.0002	0.0003 ± 0.0001
	1	0.88	1.57	1.08	1	0.92	0.69	1.08	1	1.42	0.40	0.61
HEY2	0.00004 ± 0.00001	0.00003 ± 0.00001	0.00003 ± 0.00003	0.000037 ± 0.00002	0.000028 ± 0.00001	0.000026 ± 0.00001	0.000023 ± 0.00002	0.00005 ± 0.00004	0.00003 ± 0.00001	0.00004 ± 0.00002	0.00004 ± 0.0001	0.000028 ± 0.00001
	1	0.75	0.75	0.92	1	0.93	0.82	1.79	1	1.33	1.33	0.66
NOTCH1	0.0016 ± 0.0008	0.0006 ± 0.0002	0.0007 ± 0.0006	0.0009 ± 0.0003	0.00038± 0.00007	0.0005 ± 0.0001	0.0003 ± 0.0001	0.00041 ± 0.00004	0.000038 ± 0.00002	0.000043 ± 0.00003	0.00002 ± 0.00002	0.00005 ± 0.00002
	1	0.37	0.43	0.56	1	1.32	0.79	1.08	1	1.13	0.52	1.31
NR2F2	0.2214 ± 0.0892	0.1408 ± 0.0316	0.1402 ± 0.1222	0.1667 ± 0.0003	0.1975 ± 0.0322	0.1935 ± 0.0167	0.1049 ± 0.1100	0.2387 ± 0.0763	0.3789 ± 0.1619	0.6154 ± 0.1671	0.3386 ± 0.2941	0.6618 ± 0.0772
	1	0.63	0.63	0.75	1	0.98	0.53	1.21	1	1.62	0.89	1.74
